# Use of lung ultrasound in neonates during the COVID-19 pandemic

**DOI:** 10.1590/0100-3984.2020.0110

**Published:** 2020

**Authors:** Marcia Wang Matsuoka, Silvia Maria Sucena da Rocha, Maria Augusta Bento Cicaroni Gibelli, Carla Marques Nicolau, Werther Brunow de Carvalho, Lisa Suzuki

**Affiliations:** 1 Instituto da Criança do Hospital das Clínicas da Faculdade de Medicina da Universidade de São Paulo (ICr/HC-FMUSP), São Paulo, SP, Brazil.; 2 Centro Diagnóstico do Laboratório Fleury Medicina e Saúde, São Paulo, SP, Brazil.; 3 Departamento de Pediatria da Faculdade de Medicina da Universidade de São Paulo (FMUSP), São Paulo, SP, Brazil.

**Keywords:** Ultrasonography, Lung/diagnostic imaging, Coronavirus infections/diagnostic imaging, Coronavirus, Infant, newborn, Ultrassonografia, Pulmão/diagnóstico por imagem, Infecções por coronavírus/diagnóstico por imagem, Coronavírus, Recém-nascido

## Abstract

In the current pandemic, caused by infection with severe acute respiratory syndrome coronavirus 2, ultrasound has played a fundamental role in patients who develop the resulting disease, designated coronavirus disease 2019 (COVID-19). In this study we present ultrasound images of the lungs of neonates with a suspected or confirmed diagnosis of COVID-19, distinguishing between the changes related to COVID-19 and those unrelated to the disease. Ultrasound examinations were performed by a pediatric sonographer. A total of 27 neonates were evaluated. Among those who presented no respiratory symptoms, some tested negative for COVID-19 and others tested positive. All of those who were pulmonary symptomatic, negative for COVID-19 presented transient tachypnea of the newborn and respiratory distress syndrome. Lung ultrasound images obtained in COVID-19-negative neonates showed, in some cases, a normal pattern (with A lines, few B lines, a thin, linear pleural line, and no pleural effusion), whereas in others showed coalescent B lines and areas of opacity. In two of the COVID-19-positive neonates, lung ultrasound examination showed several coalescent B lines, pleural thickening, and areas of opacity. Lung ultrasound in the neonatal period appears to be applicable within the context of the current pandemic, allowing efficient evaluation of COVID-19-related changes in neonates, as well as of pathologies inherent to the neonatal period.

## Abstract (pt)

Na pandemia atual causada pelo SARS-CoV-19, a ultrassonografia (US) tem apresentado papel fundamental nos pacientes com COVID-19. Neste trabalho são mostradas imagens ultrassonográficas de recém-nascidos (RNs) suspeitos ou positivos para COVID-19 e alterações pulmonares não relacionadas a essa doença. As imagens ultrassonográficas foram obtidas por médico especialista em US pediátrica. Foram avaliados 27 RNs, sendo incluídos RNs assintomáticos da parte respiratória, COVID negativos e positivos, e RNs sintomáticos para a parte respiratória, COVID-negativos, observados na taquipneia transitória do recém-nascido ou na síndrome do desconforto respiratório. As imagens ultrassonográficas dos RNs negativos para COVID-19 mostraram tanto o padrão normal (presença de linhas A, poucas linhas B, linha pleural fina e linear, ausência de efusão pleural) quanto a presença de linhas B coalescentes e áreas de condensação pulmonar. Destaca-se a presença de dois RNs COVID-19 positivos apresentando múltiplas linhas B coalescentes, espessamento pleural e com áreas de condensação. Com este trabalho, os autores procuram demonstrar a aplicabilidade da US pulmonar dentro do contexto da pandemia da COVID-19, incluindo as doenças inerentes ao período neonatal.

## INTRODUCTION

Since the beginning of the coronavirus disease 2019 (COVID-19) pandemic, caused by infections with the severe acute respiratory syndrome coronavirus 2 (SARS-CoV-2), much has been published about the use of lung ultrasound in the diagnosis and monitoring of adult and pediatric patients with COVID-19^(1,11,14)^. Various studies have described the alterations associated with SARS-CoV-2 infection in different age groups, at different stages of the disease, and in different organs^(2,3,14)^. Because COVID-19 is a new disease, many of those alterations have yet to be fully elucidated. In neonates in particular, studies of pulmonary changes resulting from the disease have also been carried out^(12,13)^.

The objective of this article is to disseminate lung ultrasound images obtained from neonates born of women with a suspected or confirmed diagnosis of COVID-19. The images illustrate the pulmonary changes associated with COVID-19, as well as those associated with diseases inherent to the neonatal period.

## METHOD

Lung ultrasound examinations of neonates were performed during the neonatal period. All of the neonates evaluated were born at a tertiary care hospital that had been modified during the pandemic to serve only patients with a suspected or confirmed diagnosis of COVID-19. The neonates evaluated were asymptomatic for COVID-19, being hospitalized for the usual postnatal period and receiving the clinical care they would normally receive, despite the context of the pandemic. Clinical and biochemical data were collected. A pediatric sonographer performed the ultrasound examinations, using a 7.5-10.0 MHz linear transducer and following the COVID-19 hygiene protocol established by the hospital^(4)^. Longitudinal and transversal images were obtained of the anterior, lateral and posterior chest walls.

We evaluated 27 neonates, with gestational ages at delivery ranging from 26 weeks 2 days to 38 weeks and birth weights ranging from 680 to 3,600 grams. Of the neonates who presented with no respiratory symptoms, some were COVID-19-positive and some were COVID-19-negative, whereas all of those who presented with respiratory symptoms were COVID-19-negative. In the latter group, transient tachypnea of the newborn and respiratory distress syndrome were observed.

**Figure 1:**
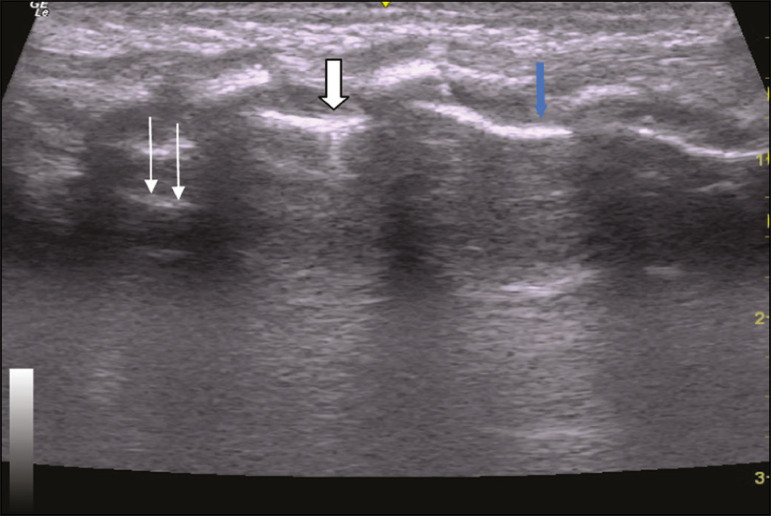
Asymptomatic COVID-19-negative neonate with normal ultrasound examination results. Presence of A lines (thin arrows), few B lines (filled arrow), regular pleural line (blue arrow).

**Figure 2:**
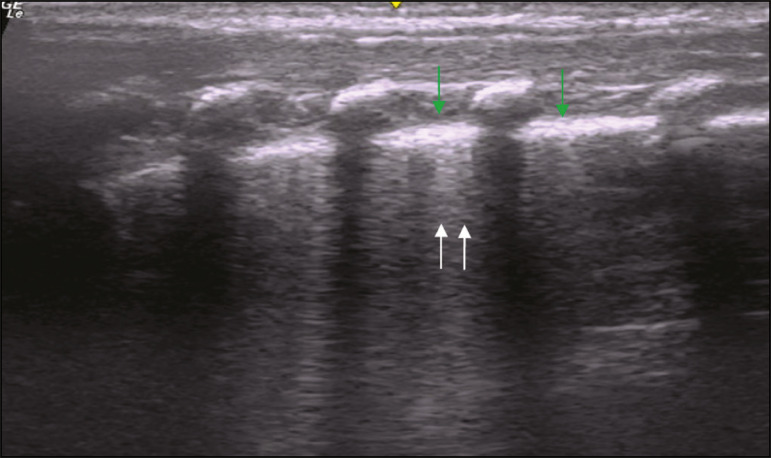
Asymptomatic COVID-19-negative neonate. Coalescent B lines (arrows) and pleural thickening (green arrows).

**Figure 3:**
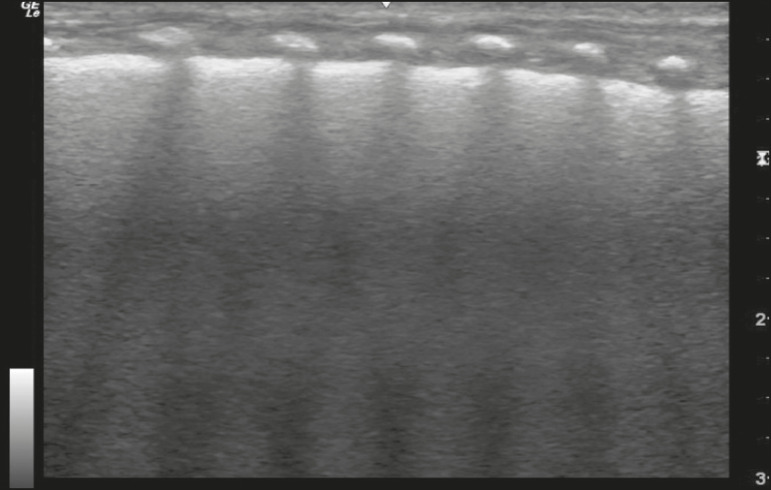
Asymptomatic COVID-19-negative neonate with transient tachypnea of the newborn. Diffuse hyperechogenicity, without A lines.

**Figure 4:**
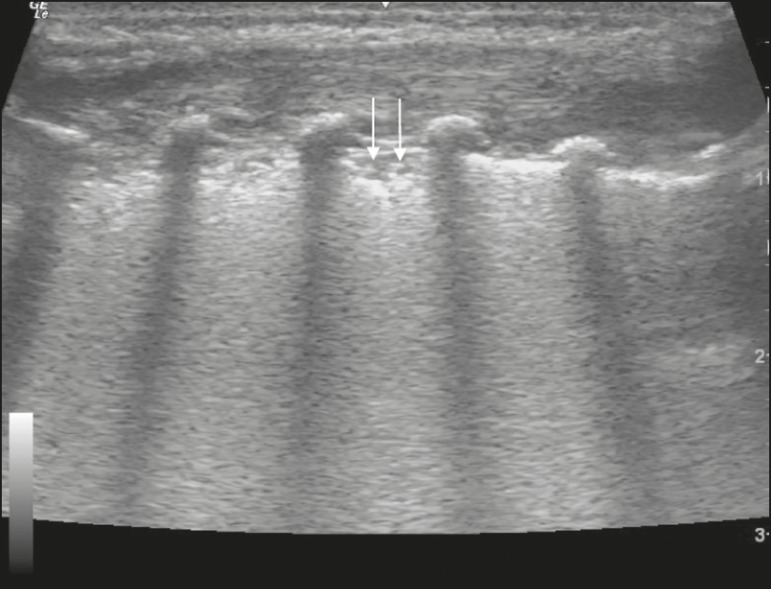
Asymptomatic COVID-19-negative neonate with respiratory distress syndrome. Pleural thickening accompanied by opacity on the lung surface and air bronchograms (arrows).

**Figure 5:**
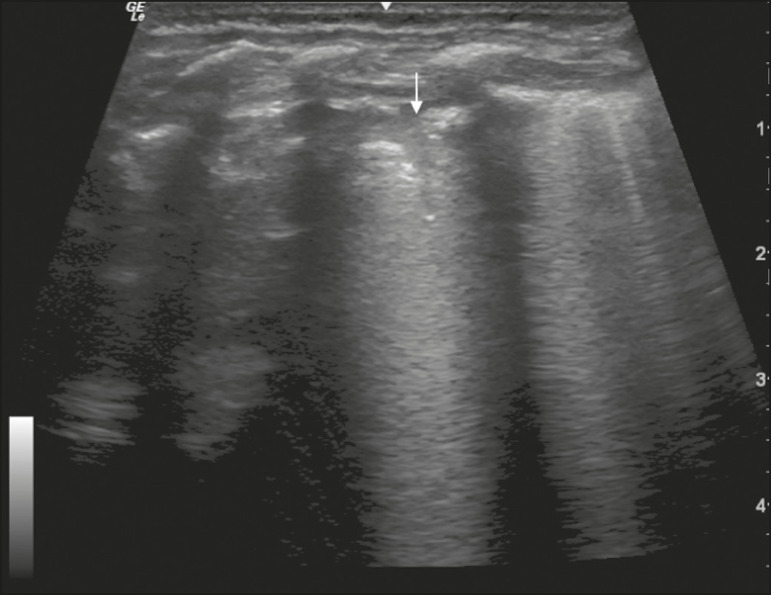
Asymptomatic COVID-19-negative neonate. Focal area of pulmonary opacity (arrow).

**Figure 6:**
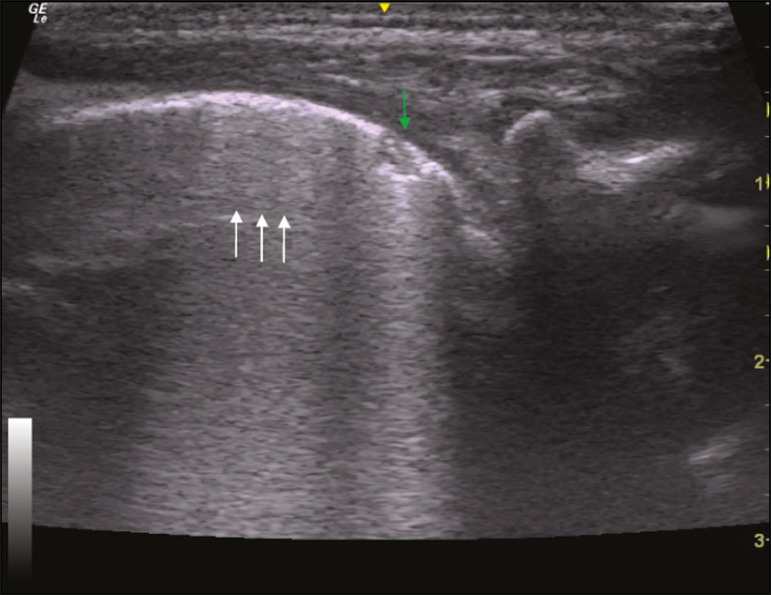
Asymptomatic COVID-19-positive neonate. Focal pulmonary opacity (green arrow) and coalescent B lines (white arrows).

**Figure 7:**
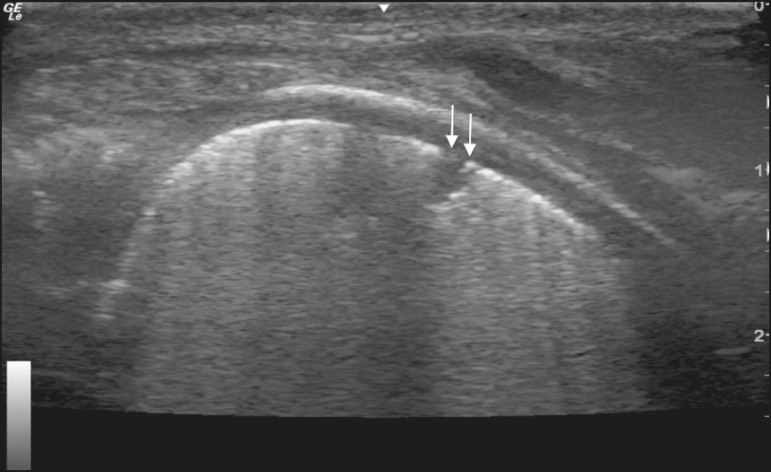
Asymptomatic COVID-19-positive neonate. Focal pulmonary opacity (arrows).

**Figure 8:**
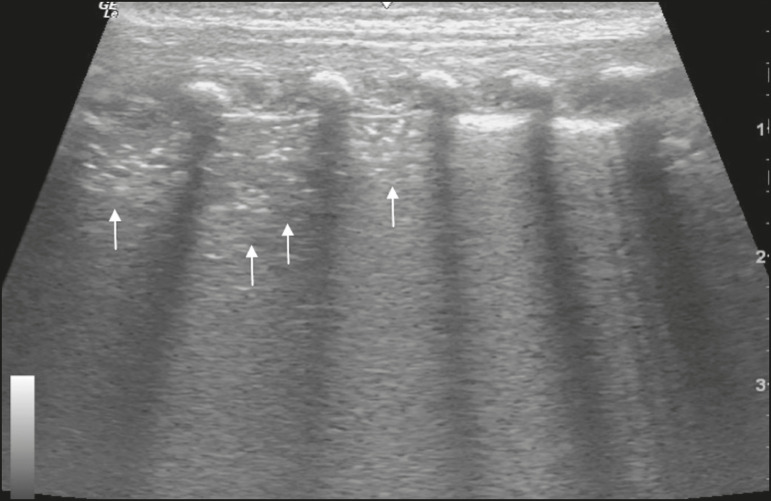
Asymptomatic COVID-19-positive neonate. Extensive area of pulmonary opacity (arrows).

In the asymptomatic COVID-19-negative neonates, the lung ultrasound findings ranged from the pattern described as normal (A lines, few B lines, a thin, linear pleural line, and no pleural effusion, as shown in Figure 1), whereas others showed coalescent B lines and pleural thickening (Figure 2); diffuse hyperechoic images throughout the lungs, without A lines (Figure 3); an irregular pleural surface with consolidation and air bronchograms (Figure 4); or areas of pulmonary condensation in the posterior chest wall (Figure 5). In the asymptomatic COVID-19-positive neonates, the lung ultrasound findings ranged from a pattern of multiple coalescent B lines, especially in the posterior lung fields, with pleural thickening and areas of pulmonary condensation (Figures 6 and 7) to one in which there was a large area of pulmonary condensation in the posterior lung field (Figure 8).

## DISCUSSION

Ultrasound has very favorable characteristics for use in pediatric patients, such as the fact that it does not expose patients to radiation and can therefore be repeated as often as necessary, as well as being widely available and noninvasive, and does not require sedation and can be performed at the bedside. Another imaging method used in the evaluation of patients with COVID-19, especially adult patients, is computed tomography (CT) of the chest. However, CT employs ionizing radiation and it should therefore be avoided or used with caution in neonates, infants, and children. In addition, CT is not always available and, when it is, neonates need to be transported, often in incubators and on ventilatory support, to the location where the CT scanner is located. In the context of the COVID-19 pandemic, the need to reduce patient transport between hospital sectors is another point in favor of the use of ultrasound^(3)^. Chest X-ray, which was employed in the first cases of COVID-19^(5)^, is less specific for the disease.

Ultrasound has been shown to play a particularly important role in the pulmonary evaluation of neonates with COVID-19, because of the characteristics of the disease, which promotes changes on the lung surface^(6,7)^. The use of ultrasound typically allows the lungs of neonates to be evaluated in a very satisfactory manner, the use of chest CT being reserved for cases in which lung ultrasound is insufficient. The small surface area of the neonate chest creates favorable conditions for the use of lung ultrasound, less extensive scans being required in neonates than in adults. In addition, the acquisition of images of the lung surface is facilitated by the fact that the chest wall is thinner in neonates.

Much has been published about the use of lung ultrasound in COVID-19. In neonates with COVID-19, the lung ultrasound changes described in the literature are the same as those observed in older children and adults^(6,7)^: absence of A lines; coalescent B lines; pleural thickening; and subpleural pulmonary pulmonary condensation.

It should be in mind that neonates have pulmonary characteristics inherent to their age, as well as physiological characteristics that vary over the course of the postnatal period. In premature neonates, lung ultrasound can show features of diseases inherent to the neonatal period or of the natural evolution occurring during that period, rather than being specifically related to COVID-19^(8)^: changes related to respiratory distress syndrome due to surfactant deficiency^(15,16)^; or changes characteristic of the first 72 h of life, when transient tachypnea of the newborn may occur^(8)^.

Studies have shown the importance of correlating clinical data with ultrasound findings in adults and children, including neonates, who for reasons still unknown have mild respiratory symptoms or remain asymptomatic when infected with SARS-CoV-2^(6,9,10)^. Unlike De Rose et al.^(6)^, who found that COVID-19-positive neonates presented with a variety of clinical conditions, ranging from respiratory symptoms to gastrointestinal symptoms, we found that all of the COVID-19-positive neonates evaluated in the present study remained asymptomatic throughout the neonatal period.

## CONCLUSION

Lung ultrasound appears to be applicable within the context of the COVID-19 pandemic. Specifically in neonates, the method is capable of identifying features of COVID-19, as well as those of diseases inherent to the neonatal period.
